# Editorial: Using novel technologies and models to identify biomarkers and explore therapeutic strategies for neurological disorders

**DOI:** 10.3389/fnbeh.2023.1151667

**Published:** 2023-03-23

**Authors:** Liwei Xing, Chengbiao Wu, Jiaojian Wang, Sheng Wei, Kai Yuan, Dongdong Qin

**Affiliations:** ^1^School of Basic Medical Sciences, Yunnan University of Chinese Medicine, Kunming, Yunnan, China; ^2^Department of Neurosciences, University of California, San Diego, San Diego, CA, United States; ^3^State Key Laboratory of Primate Biomedical Research, Institute of Primate Translational Medicine, Kunming University of Science and Technology, Kunming, Yunnan, China; ^4^Experimental Center, Shandong University of Traditional Chinese Medicine, Jinan, China; ^5^The Second Clinical Medical School, Yunnan University of Chinese Medicine, Kunming, Yunnan, China

**Keywords:** neurological disorders, biomarker, therapeutic strategy, animal model, novel technology

Neurological disorders are a group of diseases that affect the structure or function of the central and peripheral nervous systems. In addition to stroke and diseases of the blood vessels supplying the brain, representative central nervous system diseases also include neurodevelopmental disorders brought on by faulty genes, risky environmental factors, or gene-environment interactions, such as autism spectrum disorders (ASD), neurodegenerative disorders where nerve cells are damaged or die, such as Parkinson's disease (PD) and Alzheimer's disease (AD), as well as major depressive and bipolar disorders (Pena et al., 2020). More than 600 neurological disorders afflict the nervous system (Matilla-Dueñas et al., 2017). The absolute number of deaths brought by neurological disorders has climbed by 39%, and disability-adjusted life years have increased by 15% over the past 30 years (Feigin et al., 2020). According to a systematic analysis for the Global Burden of Disease Study 2015, neurological diseases are the second most common cause of death (GBD 2015 Disease Injury Incidence Prevalence Collaborators, 2016). Neurological disorders have attracted increasing attention from around the world and the resulting burden has become more widely acknowledged as a global public health concern in the coming decades.

Although these neurological disorders place a significant burden on society and individuals, the pathogenesis and biomarkers of these diseases are not yet fully understood. For example, ASD is a complex neurodevelopmental disorder probably caused by several pathological factors, such as neurochemical alterations including changes of gamma aminobutyric acid, glutamate, serotonin, dopamine, N-acetyl aspartate, oxytocin, arginine-vasopressin, melatonin, vitamin D, orexin, endogenous opioids, and acetylcholine (Marotta et al., 2020), abnormal brain structure and development (Gibbard et al., 2018; Lee et al., 2020; Thompson et al., 2020), as well as immunity dysregulation (Kim et al., 2017; Robinson-Agramonte et al., 2022). Studies have also shown that more than 1,000 genes are related to the pathogenesis of ASD (Famitafreshi and Karimian, 2018; Qin et al., 2021). At the same time, environmental factors, including nutrition, medications, toxic substances, and maternal infections during pregnancy, have been extensively studied and found to be associated with ASD (Wang et al., 2016). The etiology and pathogenesis of depression are reported to be highly associated with synaptic remodeling, transcription factors and epigenetics, immunity and inflammation, as well as astrocyte function (Ménard et al., 2016). However, the understanding of the mechanisms and biomarkers underlying these neurological disorders has been still far from complete.

Based on public health trends and epidemiology patterns, the World Health Organization (WHO) has proposed a recommended formulary for high-priority diseases, serving as a guide for countries, particularly low- and lower-middle-income countries, to develop their own national essential medicines list. However, drugs that target neurologic disorders are poorly represented on the WHO model list (Rimmer et al., 2017). Besides, behavioral therapy, neurostimulation, and dietary interventions are also recommended in therapies and preventive measures of neurological disorders. New targeted drugs and novel therapeutic strategies concerning different pathogenesis are still depending on sufficient clinical and pre-clinical trials.

In view of the above realization, this special issue was organized to advance the understanding of the pathogenesis and biomarkers of neurological disorders, as well the most recent and advanced work on novel technologies identifying or preventing neurological disorders. Furthermore, novel therapeutic strategies are also discussed. This will serve as a foundation and perhaps provide valuable hints for clinical therapies and pharmacological development. For this Research Topic, twelve manuscripts have been submitted. After 7 months of critical peer review, ten papers have been accepted.

In the original research titled “*Brain somatic variant in Ras-like small GTPase RALA causes focal cortical dysplasia type II*”, Xu et al. performed deep whole-exome sequencing and targeted amplicon sequencing in the postoperative brain tissue of epilepsy patients with focal cortical dysplasia type II (FCD II). In their study, HEK293T cells were transfected *in vitro*, with wild-type and mutant RALA plasmids were transfected into the local cortex of mice using *in utero* electroporation to evaluate the effect of RALA c.G482A on neuronal migration. The results demonstrated that the somatic gain-of-function variant of RALA activated the mTOR pathway and led to neuronal migration disorders in the brain, facilitating the development of FCD II.

In the case report titled “*Case report: Identification and clinical phenotypic analysis of novel mutation of the PPP1CB gene in NSLH2 syndrome*”, He X. et al. screened and analyzed the genetic mutations in a patient with Noonan syndrome with loose anagen hair-2 (NSLH2) in Yunnan Province, China. The clinical manifestations of NSLH2 included prominent forehead, yellowish hair, slightly wide eye distance, sparse eyebrows, bilateral auricle deformity, reduced muscle tension, as well as cardiac and visual abnormalities. This article identified a novel mutation of PPP1CB, which enriched the mutation spectrum of the PPP1CB gene and provided a basis for the diagnosis of NSLH2.

In the methods article titled “*Development and validation of a system for the prediction of challenging behaviors of people with autism spectrum disorder based on a smart wearable shirt: A mixed-methods design*”, Zwilling et al. developed an ML algorithm, which was capable of predicting immediate challenging behavior (CB) occurrence based on physiological parameter variations. An efficient proof of concept (POC) was also carried out to identify the strengths and weaknesses of the developed system. The results demonstrated the developed algorithm could be used to predict CBs that were about to occur in the upcoming 1 min.

In the mini review titled “*Research progress on transcranial magnetic stimulation for post-stroke dysphagia*”, Li Y. et al. discussed the effectiveness, mechanisms, potential limitations, and prospects of transcranial magnetic stimulation (TMS) for clinical application in post-stroke dysphagia rehabilitation. This has introduced a safe and non-invasive technology of nerve stimulation that can be used to directly manipulate post-stroke dysphagia.

In the review titled “*Transcranial direct current stimulation of the dorsolateral prefrontal cortex for treatment of neuropsychiatric disorders*”, Li Q. et al. performed searches on PubMed to collect clinical and preclinical studies that using transcranial direct current stimulation (tDCS) as neuromodulation technique, dorsolateral prefrontal cortex (DLPFC) as the stimulation target in treating neuropsychiatric disorders. The results indicated that tDCS stimulation of DLPFC could alleviate the clinical symptoms of schizophrenia, depression, drug addiction, attention deficit hyperactivity disorder and other mental disorders.

In the original research titled “*Safety and effects of transcranial direct current stimulation on hand function in preschool children with hemiplegic cerebral palsy: A pilot study*”, He W. et al. designed a crossover, single-blind, sham-controlled study in 30 preschool children with hemiplegic cerebral palsy (HCP). Transcranial direct current stimulation (tDCS) on the primary motor cortex of the affected hemisphere was given with a 24-h interval between the two sessions. Box and Block Test, Selective Control of the Upper Extremity Scale, Modified Ashworth Scale, and Melbourne Assessment 2 were conducted at baseline, immediately, and 90 min after each session. The results supported the safety and efficacy of a single anodal tDCS on improving the manual dexterity of the hemiplegic hand for preschool children with HCP.

In the mini review titled “*Application of cognitive bias testing in neuropsychiatric disorders: a mini-review based on animal studies*”, Zhang et al. summarized the application of cognitive bias tests in animal models of neuropsychiatric disorders such as depression, anxiety, bipolar disorder, and pain. They also discussed its critical value in the identification of neuropsychiatric disorders and the validation of therapeutic approaches.

In the review titled “*Research progress on the role of vitamin D in autism spectrum disorder*”, Wang et al. reviewed the correlation between vitamin D level and ASD, the effects of vitamin D supplementation on ASD, the possible mechanism of vitamin D involved in ASD, and insights from ASD animal models. This can help to open-up a simple, cheap, and safe strategy for the prevention and treatment of ASD.

In the original research titled “*The effect of constraint-induced movement therapy combined with repetitive transcranial magnetic stimulation on hand function in preschool children with unilateral cerebral palsy: A randomized controlled preliminary study*”, Wu et al. designed a prospective, assessor-blinded, randomized controlled study. In their study, 40 preschool children (aged 2.5–6 years) with unilateral cerebral palsy (UCP) were randomized to receive 10 days of constraint-induced movement therapy (CIMT) combined with active or sham rTMS (repetitive transcranial magnetic stimulation). Upper limb extremity, social life ability, and perceived changes by parents and motor-evoked potentials were assessed. The CIMT plus active rTMS had greater gains in the affected hand function (range of motion, accuracy, and fluency) than the CIMT plus sham rTMS group, but there was no significant difference in muscular tone, social life ability, and perceived changes by parents between the two groups. This demonstrates that the treatment of CIMT combined with rTMS is safe and feasible for preschool children with UCP.

In the review titled “*Research progress on rheumatoid arthritis-associated depression*”, Liu et al. provided an overview of the etiology and pathological mechanisms of rheumatoid arthritis-associated depression. They also reviewed recent advances in treatment with biologics, which would facilitate the development of new and effective prevention and treatment strategies.

Overall, these studies have systematically explored the pathophysiology and biomarkers of neurological disorders. They also covered the most recent and cutting-edge research on technologies for diagnosing or preventing neurological disorders, as well as novel therapy approaches. Future large-scale multi-center randomized controlled trials and in-depth mechanistic analysis are still required to further clarify the pathophysiological mechanisms underlying neurological disorders, thereby promoting translational medicine and drug development.

## Author contributions

All authors listed have made a substantial, direct, and intellectual contribution to the work and approved it for publication.
